# Analysis of metabolic characteristics in a rat model of chronic pancreatitis using high-resolution magic-angle spinning nuclear magnetic resonance spectroscopy

**DOI:** 10.3892/mmr.2014.2738

**Published:** 2014-10-22

**Authors:** BING TIAN, CHAO MA, JIAN WANG, CHUN-SHU PAN, GEN-JIN YANG, JIAN-PING LU

**Affiliations:** 1 Department of Radiology, Changhai Hospital of Shanghai, Shanghai 200433, P.R. China; 2 Pharmaceutical Analysis and Testing Center, School of Pharmacy, The Second Military Medical University, Shanghai 200433, P.R. China

**Keywords:** chronic pancreatitis, grading, metabolism, nuclear magnetic resonance, fibrosis

## Abstract

Pathological and metabolic alterations co-exist and co-develop in the progression of chronic pancreatitis (CP). The aim of the present study was to investigate the metabolic characteristics and disease severity of a rat model of CP in order to determine associations in the observed pathology and the metabolites of CP using high-resolution magic-angle spinning nuclear magnetic resonance spectroscopy (HR-MAS NMR). Wistar rats (n=36) were randomly assigned into 6 groups (n=6 per group). CP was established by administering dibutyltin dichloride solution into the tail vein. After 0, 7, 14, 21, 28 and 35 days, the pancreatic tissues were collected for pathological scoring or for HR-MAS NMR. Correlation analyses between the major pathological scores and the integral areas of the major metabolites were determined. The most representative metabolites, aspartate, betaine and fatty acids, were identified as possessing the greatest discriminatory significance. The Spearman’s rank correlation coefficients between the pathology and metabolites of the pancreatic tissues were as follows: Betaine and fibrosis, 0.454 (P=0.044); betaine and inflammatory cell infiltration, 0.716 (P=0.0001); aspartate and fibrosis, −0.768 (P=0.0001); aspartate and inflammatory cell infiltration, −0.394 (P=0.085); fatty acid and fibrosis, −0.764 (P=0.0001); and fatty acid and inflammatory cell infiltration, −0.619 (P=0.004). The metabolite betaine positively correlated with fibrosis and inflammatory cell infiltration in CP. In addition, aspartate negatively correlated with fibrosis, but exhibited no significant correlation with inflammatory cell infiltration. Furthermore, the presence of fatty acids negatively correlated with fibrosis and inflammatory cell infiltration in CP. HR-MAS NMR may be used to analyze metabolic characteristics in a rat model of different degrees of chronic pancreatitis.

## Introduction

Chronic pancreatitis is predominantly characterized by extensive and permanent fibrosis in the basic structure of the pancreas, which is a pathological process with major features of ductal alterations, fibrosis surrounding the pancreatic lobule and final scar formation in the pancreatic lobule (1–3). Pathological and metabolic alterations co-exist and co-develop in the disease progression of chronic pancreatitis. Detection of characteristics of the metabolite levels may facilitate a greater understanding of the pathophysiological events and aid in the clinical treatment of the disease (4).

High-resolution magic-angle spinning nuclear magnetic resonance spectroscopy (HR-MAS NMR) is a non-invasive method which can be used to analyze the metabolic components of *in vitro* tissues, and it exhibits high sensitivity and accuracy in analyzing the components and concentrations of different compounds in tissues (5–8).

In the current study, a rat model of CP was established and the development of this disease was studied at different time points. HR-MAS NMR of pancreatic tissues was performed *in vitro*, in order to determine the metabolic profiles of CP at different time periods in the rat model. In addition, correlations between the observed pathological changes in pancreatic tissues and changes in metabolite profiles were assessed. The major aim of studying any observed correlation was to identify a non-invasive and effective approach for determining the major pathological characteristics of CP.

## Materials and methods

### Establishing a rat model for CP

The present study was approved by the local Animal Ethics Committee of the Shanghai Changhai Hospital Ethics Committee (Shanghai, China). A total of 36 male Wistar rats, each weighing ~200 g, were purchased from the Laboratory Animal Center of the Second Military Medical University (Shanghai, China). The rats were maintained in environmentally controlled conditions (temperature, 22±2°C; light/dark cycle, 12/12 h; relative humidity, 60–70%). All rats were randomly assigned to 6 groups with 6 rats per group. Dibutyltin dichloride (DBTC; Sigma-Aldrich, St Louis, MO, USA) was dissolved in 100% ethanol, and mixed with glycerol (at a ratio of ~1:2:3). DBTC (~200 μl) was administered to each rat by tail vein injection at a dose of 8 mg/kg (9) to establish CP.

One group of rats was sacrificed on each of the following days after establishing the disease model: 0 (immediate sacrifice after establishing the model of CP), 7, 14, 21, 28 and 35. The rats were anesthetized with ~0.2 ml of pentobarbital solution (Sigma-Aldrich) by intraperitoneal injection, and then the abdomen was exposed. In total, 30 pancreatic tissue specimens were obtained from the successful models. The pancreatic tissues were observed, and carefully separated. Immediately following this initial observation, a number of the pancreatic tissue specimens were placed in 4% formaldehyde solution for pathological examination, and other tissue specimens were snap-frozen in liquid nitrogen and immediately stored at −80°C for HR-MAS NMR analysis.

### Pathological examination and scoring

Formaldehyde-fixed pancreatic tissues were embedded in paraffin, sliced into sections and stained with hematoxylin and eosin (HE; Beyotime Institute of Biotechnology, Haimen, China). The pancreatic tissue sections were scored by a previously described method (10–12). The sections were examined for abnormal structures, tubular complex, gland atrophy, fibrosis, edema and inflammatory cell infiltration, which collectively served as basic indicators of disease pathology. The pathological sections were scored (Table I) according to the following scoring system: 0 (no abnormal structure); 1 (mild, <10%); 2 (moderate, 10–50%); 3 (severe, >50%).

### HR-MAS NMR

Snap-frozen pancreatic tissues were thawed at room temperature, and the central specimens, each weighing ~15 mg, were carefully cut on sterile dry culture dishes. The tissue specimens were washed with deuterated water and transferred to 4-mm ZrO_2_ tubes (Unipretec Ceramic Technology Co., Ltd., Xiamen, China) with a spherical cavity. The specimens were next placed in a Bruker Avance III 600 spectrophotometer (Bruker BioSpin, Rheinstetten, Germany) with a standard HR-MAS probe for NMR measurements.

One-dimensional ^1^H NMR spectroscopy was performed using Carr-Purcell-Meiboom-Gill (CPMG) pulse sequences with pre-saturation of the water peak, with a total echo time of 180 msec. The signals of macromolecules and certain metabolites with a short spin-spin (T2) relaxation time in the tissues were suppressed. Other parameters included: ^1^H resonance frequency, 600.13 MHz; magic-angle spinning speed, 5 kHz; test temperature, 300.0 K; spectral width, 12.02 kHz; and recovery time, 2 sec. The spectral peaks of the major metabolites were identified based on observations from previous studies (8,9).

A specific software program enabled Fourier transform of the experimental data (TopSpin version 3.0; Bruker BioSpin) and the corresponding phase and baseline correction were also completed for this data set. The data obtained from one-dimensional proton CPMG experiments were calibrated using the fractional anisotropy peak at 1.31 ppm, with a range of 0.5–4.6 ppm. Principal component analysis (PCA) was employed to analyze the experimental data set using the AMIX software program (version 3.9.7; Bruker BioSpin), and the relative concentrations of the typical metabolites were calculated. The corresponding integral areas in the CPMG spectra were obtained for statistical analysis.

### Statistical analysis

The integral areas corresponding to the major metabolites in the CPMG spectrum of the three groups were entered into an Excel spreadsheet (Microsoft Corporation, Redmond, WA, USA). The integral areas of the major metabolites, which were screened by PCA, were further studied by multiple rank order logistic regression analysis in order to screen the metabolites with the most discriminatory value. Spearman rank-order correlation analysis of the scoring results of fibrosis with inflammatory cell infiltration and the integral areas of the major metabolites was also performed. All statistical analyses were performed with the SPSS statistical software program, version 16.0 (SPSS, Inc., Chicago, IL, USA). P<0.05 was considered to indicate a statistically significant difference.

## Results

### Pathological scores

One day after establishing CP in the rat model, two rats (one from the day 7 group and the other from the day 28 group) died. Four additional rats died two days after establishing the CP model (two from the day 21 group and two from the day 35 group).

**Figure 1:**
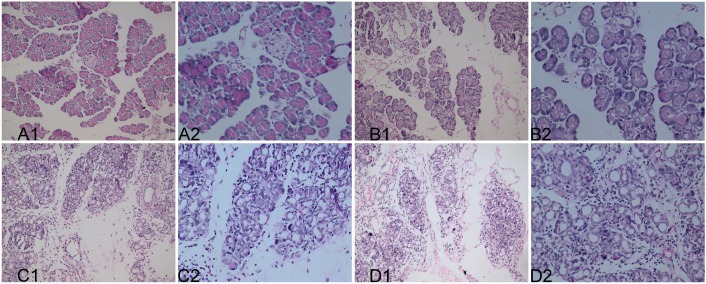
Pathological examination of pancreatic tissues by hematoxylin and eosin staining. (A) Day 7 group tissues. Slight atrophy of the pancreatic gland with infiltration by low frequencies of inflammatory cells and fibrosis. Total score = 5; fibrosis score = 1; and inflammatory cell infiltration score = 1. (B) Day 14 group tissues. Clear atrophy of the pancreatic gland and moderate edema of the pancreatic tissues, with inflammatory cell infiltration and fibrosis. Total score = 7; fibrosis score = 2; and inflammatory cell infiltration score = 2. (C) Day 21 group tissues. Clear atrophy of the pancreatic gland and moderate edema of the pancreatic tissues, with infiltration with relatively high numbers of inflammatory cells and fibrosis. Total score = 7; fibrosis score = 2; and inflammatory cell infiltration score = 2. (D) Day 35 group tissues. Clear atrophy of the pancreatic gland and evident edema of the pancreatic tissues, with infiltration with high numbers of inflammatory cells and fibrosis. Total score = 11; fibrosis score = 2; and inflammatory cell infiltration score = 3. A1-D1, magnification ×200; A2-D2, magnification ×400.

The pathological scores of non-normal structures, tubular complex, gland atrophy, fibrosis, edema, and inflammatory cell infiltration are shown in Table II. The extent of fibrosis and inflammatory cell infiltration was aggravated according to the increased duration of CP. Based on the examination of the pathological specimens, the CP model was classified into three groups: A control group (n=10, six from the day 1 group, three from the day 35 group and one from the day 28 group), a mild CP group (n=10, five from each of the day 7 and 14 groups) and a severe CP group (n=10, four from each of the day 21 and 28 groups, and one from each of the day 14 and 35 groups). Pathological examination of the pancreatic tissues following HE staining is shown in Fig. 1.

### Pancreatic tissue analysis

**Figure 2:**
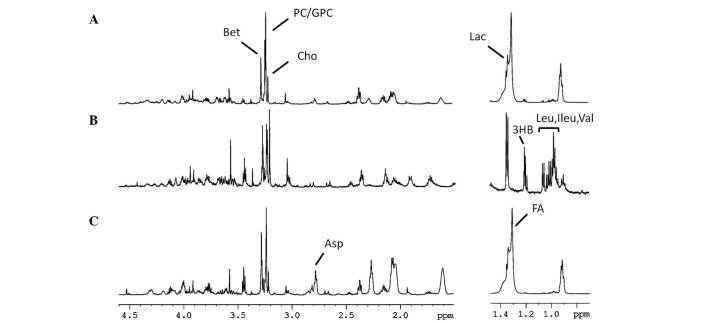
Representative ^1^H MAS NMR spectra of the pancreatic tissues in the (A) control, (B) severe chronic pancreatitis and (C) mild chronic pancreatitis groups. The region of δ1.50–4.60 ppm is zoomed in 4 times of that in δ0.50–1.50 ppm (600 MHz, CPMG pulse sequence, temperature 300.0 K). Bet, betaine; PC/GPC, phosphocholine/glycerophosphocholine; Cho, choline; Lac, lactate; 3HB, 3-hydroxybutyrate; Leu, Ileu, val, leucine/isoleucine/valine; Asp, aspartate; FA, fatty acids; MAS, magic-angle spinning; NMR, nuclear magnetic resonance; CPMG, Carr-Purcell-Meiboom-Gill.

**Figure 3:**
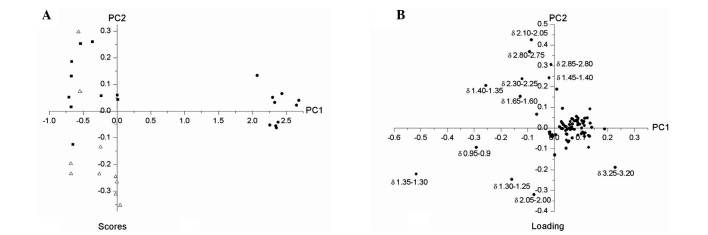
Principal components analysis (A) scores plots and (B) loadings plots. From the data of 30 ^1^H CPMG NMR spectra (δ0.50–4.60 ppm). ^n^, control group; ^Δ^, mild chronic pancreatitis group; and ^l^, severe chronic pancreatitis group). CPMG, Carr-Purcell-Meiboom-Gill; NMR, nuclear magnetic resonance.

Following the analysis of pancreatic tissue specimens obtained from the control, mild and severe CP groups by HR-MAS (Fig. 2), PCA was employed to screen 8 metabolic biochemicals including betaine, phosphocholine/glycerophosphocholine, choline, aspartate, lactate, fatty acids, 3-hydroxybutyrate and isoleucine/leucine/valine (Fig. 3), and the scores from each of the models within the three CP groups formed three distinct clusters (Fig. 3A).

Multiple rank-order logistic regression analysis of integral areas within the major metabolites is shown in Table III. According to the descending order of the regression coefficient values (estimated values) of the major metabolites obtained from logistic regression analysis, the most important metabolites to discriminate the severity in CP were aspartate, betaine and fatty acids.

### Correlation analysis

The correlation analysis between the pathology grading scores of fibrosis and inflammatory cell infiltration and the related integral area of main metabolites in CP are shown in Table IV. The content of betaine shows positive correlation with fibrosis (correlation coefficient, 0.454; P=0.044) and inflammatory cell infiltration (correlation coefficient, 0.716; P=0.0001). The content of aspartate shows negative correlation with fibrosis (correlation coefficient, −0.768; P=0.0001). No significant correlation between the content of aspartate and inflammatory cell infiltration was found (correlation coefficient, −0.394; P=0.085). In addition, the content of fatty acids shows negative correlation with fibrosis (correlation coefficient, −0.764; P=0.0001) and with inflammatory cell infiltration (correlation coefficient, −0.619; P=0.004).

## Discussion

In the present study, rat models of CP with different severity were established by tail vein injection of DBTC solution. It was found that inflammatory cell infiltration and fibrosis exhibit vital roles in the progression of CP. According to the standard scoring criteria of pathological sections, thirty rats were divided into three groups, with 10 rats in each group. For the mild CP group, all of the pathology specimens were collected from the day 7 and 14 groups, while 9 specimens were obtained from the day 21, 28 and 35 groups for the severe CP group. According to the pathological results, it is reasonable to conclude that the extent of fibrosis and inflammatory cell infiltration causes an increase with the duration of CP.

In current approaches for scoring the pathology of CP, the extent of inflammatory cell infiltration is commonly identified by counting the mean number of inflammatory cells in five high-powered fields of view (13,14). Inflammatory cell infiltration and fibrosis exhibit vital roles in the development of CP. Pancreatic fibrosis is an important pathophysiological process, which affects the prognosis of CP (15). Thus, an assessment of the occurrence, extent and development of fibrosis can identify the duration and prognosis of CP. The most commonly used method in the pathological evaluation of fibrosis in CP is van Gieson staining of pancreatic collagen fibers, followed by scoring the mean area of collagen fiber staining in five high-powered fields of view (16). Pathological examination is the most fundamental method for diagnosis of numerous diseases. However, biopsy of the pancreas is difficult to perform and carries a certain risk. Pathological examination is challenging to perform for atypical early-stage CP. Therefore, there remains an urgent requirement for a simple and effective method of assessing fibrosis and inflammatory cell infiltration.

Magnetic resonance spectroscopy (MRS) has high sensitivity and resolution, allows *in vitro* testing of metabolites, and has been widely used in the field of metabolomics (17). With the rapid development of MRS techniques, high field-strength equipment gradually emerged, and with it the sensitivity of MRS continuously improved. This enabled a marked increase in the application of MRS in clinical diagnostics and basic laboratory studies (18). Currently, the clinical application of the 1.5T or 3.0T magnetic resonance imaging (MRI) scanner can directly perform MRS analyses of pancreatic tissues and lesions *in vivo*. However, this approach suffers from the drawbacks of low magnetic field strength and low spatial resolution, and as a consequence, the interference of respiratory and motion artifacts of other abdominal organs, which collectively dampen the available information of the metabolites studied. Thus, it is necessary to conduct a study in rat models, so that the metabolic profiling of *in vitro* tissues is undertaken prior to any clinical application or intervention (19). In the current study, HR-MAS NMR was used to investigate pancreatic tissues obtained from rats with CP, and separate the various components of different amino acids. In the use of this approach, physical or chemical treatment of the samples is not necessary, small specimen sizes are all that is required (10–20 mg) and complete placement (100%) of the sample in the detection coils is possible. Consequently, HR-MAS NMR ensures even magnetic exposure of the sample and improved sensitivity and resolution. This study in the field of metabolomics is auxiliary to proteomics, and goes a step further in the study of pancreatopathy.

Spearman’s rank correlation, a rank correlation test, is a statistical approach used to measure bivariate data sets and rank that data for linear correlation analysis. The Spearman’s rank correlation between fibrosis and inflammatory cell infiltrates in 20 cases of CP and their metabolites aspartate, betaine and fatty acids in the present study demonstrated the utility of this approach.

Betaine is a highly efficient active methyl donor. During the process of tumor occurrence, low overall methylation and locally high levels of methylation of DNA in tumor cells are observed. It has been found that the level of betaine in pancreatic cancer tissues is markedly reduced as compared with that in normal pancreatic tissues. In the current study, it was shown that betaine positively correlated with both fibrosis and inflammatory cell infiltration of CP. The common form of aspartate is L-aspartate, which can provoke disorders in protein synthesis through hydrolyzing asparagines and thereby inhibit the growth and proliferation of tumor cells. L-aspartate rarely induces acute pancreatitis, which is the most severe adverse reaction associated with its use. However, once acute pancreatitis occurs, it progresses rapidly and the condition is severe, with morbidity occurring quite rapidly due to the occurrence and consequences of shock (20). It is thought that L-aspartate directly damages pancreatic acini, which leads to effusion, activation and autodigestion of pancreatic cells, the corollary of which promotes the emergence of acute pancreatitis (21).

Determination of serum unsaturated fatty acid levels reveals that fatty acids may be important components in the complex network of the core systemic inflammatory response syndrome associated with the onset of acute and severe CP. However, the role of fatty acids in CP remains unclear. The present study showed that fatty acids were negatively correlated with the fibrosis and inflammatory cell infiltration observed in CP.

The extent of fibrosis and inflammatory cell infiltration exhibits an aggravated tendency to increase with the duration of CP. Discrimination screening of the metabolites using HR-NMR indicated the importance of betaine, phosphocholine/glycerophosphocholine, choline, aspartate, 3-hydroxybutyrate, lactate, fatty acids, and isoleucine/leucine/valine. From these metabolites, the three identified as possessing the greatest discriminatory significance were found to be aspartate, betaine and fatty acids. The metabolite betaine positively correlated with fibrosis and inflammatory cell infiltration in CP. In addition, aspartate negatively correlated with fibrosis in CP, but exhibited no significant correlation with inflammatory cell infiltration in CP. Furthermore, the presence of fatty acids negatively correlates with fibrosis and inflammatory cell infiltration in CP. HR-MAS NMR may be used to analyze metabolic characteristics in a rat model of different degrees of chronic pancreatitis.

## Figures and Tables

**Table I:** Scoring criteria of pathological sections of pancreatic specimens *in vitro.*

Degree	Abnormal structure	Gland atrophy	Fibrosis	Edema	Inflammatory cell infiltration	Tubular complex
None	0	0	0	0	0	0
Mild <10%	0	1	1	1	1	1
Moderate 10–50%	0	2	2	2	2	2
Severe >50%	0	3	3	3	3	3

**Table II:** Pathological scoring of the pancreatic tissues after different time periods (mean ± standard deviation).

	Day					
	
Pathology	0	7	14	21	28	35
Abnormal structure	0.00±0.00	1.00±0.63	1.20±0.44	2.00±1.00	2.33±0.58	2.75±0.50
Tubular complex	0.00±0.00	0.00±0.00	0.00±0.00	0.00±0.00	0.00±0.00	0.00±0.00
Gland atrophy	0.00±0.00	1.00±0.00	1.00±0.00	2.00±1.00	2.33±0.58	2.25±0.50
Fibrosis^a^	0.00±0.00	1.00±0.00	1.00±0.00	2.33±1.15	1.67±0.58	2.75±0.96
Edema	0.00±0.00	1.00±0.00	1.00±0.00	2.67±0.58	2.33±0.58	2.75±0.96
Inflammatory cell infiltration^a^	0.00±0.00	1.00±0.00	1.00±0.00	2.33±0.58	2.00±1.00	1.50±0.58
Total score	0.00±0.00	5.00±0.71	5.20±0.44	11.33±1.15	10.66±0.57	11.00±0.81

a Index selected for analysis in the current study.

**Table III:** Multiple rank order logistic regression analysis of the major metabolites in the control, mild and severe chronic pancreatitis groups.

	Betaine	Phosphocholine/Glycerophosphocholine	Choline	Aspartate	Lactate	Fatty acid	3-Hydroxybutyrate	Isoleucine/Leucine/Valine
Estimate^a^	−3.477	−0.372	−0.296	3.773	0.401	−0.983	−1.401	−0.629
P-value	0.017	0.564	0.854	0.001	0.248	0.004	0.060	0.274

a Estimate indicates multiple rank order logistic regression coefficients.

**Table IV:** Spearman’s rank correlation tests between the pathology and metabolites of the pancreatic tissues (n=20).

	Score of fibrosis		Score of inflammatory cell infiltration	
		
Metabolite	Correlation coefficient	P-value	Correlation coefficient	P-value
Betaine	0.454	0.0440	0.716^a^	0.0001
Aspartate	−0.768^a^	0.0001	−0.394	0.0851
Fatty acid	−0.764^a^	0.0001	−0.619^a^	0.0040

a Two-sided P<0.01.
